# Effects of Different Selenium Concentrations on Agronomic Traits, Antioxidant Defense, and Leaf Metabolome in Blueberry (*Vaccinium corymbosum* L. ‘Brigitta’)

**DOI:** 10.3390/plants15101532

**Published:** 2026-05-17

**Authors:** Keqin He, Siyu Wang, Yi Zhou, Yihang Liu, Guangrong Cui, Hao Xia

**Affiliations:** 1 College of Resources and Environment, Anhui Science and Technology University, Chuzhou 233100, China; hekq@ahstu.edu.cn (K.H.); wsy1002816@163.com (S.W.); zhouy@ahstu.edu.cn (Y.Z.); liuyihang0208@163.com (Y.L.); 2 The Industrial Crop Institute, Anhui Academy of Agricultural Sciences, Hefei 230001, China

**Keywords:** antioxidant enzyme activities, blueberry, fruit quality, leaf metabolites, selenium level

## Abstract

Selenium (Se) is an important micronutrient that is required in very small amounts and plays a significant role in enhancing plant growth, stress resistance, and fruit quality. In this study, we investigated the effects of different sodium selenite concentrations (CK, 0 mg/L; Se1, 0.50 mg/L; Se2, 1.00 mg/L, Se3, 2.00; and Se4, 3.00 mg/L) on the growth, nutrient absorption, antioxidant capacity, and leaf metabolome of blueberry (*Vaccinium corymbosum* L. ‘Brigitta’) in hydroponic culture. Our results showed that moderate Se concentration (1.00 mg/L, Se2) had noticeable enhancements in key traits like taller plants, thicker stems, a greater number of leaves, and stem fresh weight, with increases of 60.23%, 61.90%, 36.05%, and 87.97%, respectively, compared to the CK. In addition, the appropriate application of Se fertilizer (1.0 mg/L, Se2) can enhance the absorption of macronutrients by plants, with the total contents of nitrogen (N), phosphorus (P), and potassium (K) increasing by 48.11%, 15.85%, and 14.25%, respectively, compared to CK. In comparison to CK, the content and accumulation of total Se rose dramatically under the Se4 treatment, showing increases of 2300% and 2514%. The contents of chlorophyll and antioxidant enzyme activities were maximized at Se2, while excessive Se (Se4) led to oxidative damage, as indicated by elevated MDA, H_2_O_2_, and O_2_^−^ levels. Moreover, metabolomic analysis revealed that moderate Se concentration (Se2) significantly altered metabolic pathways related to aminoacyl-tRNA biosynthesis, arachidonic acid metabolism, and ABC transporters, with downregulation of key metabolites in sugar and organic acid metabolism (e.g., α-D-glucose-6-phosphate, L-lactic acid, maleic acid). In contrast, high Se concentration (Se4) disrupted these pathways and promoted volatile compound accumulation. These findings demonstrate that moderate Se application enhances blueberry growth and quality by regulating nutrient uptake, antioxidant defense, and primary metabolism, whereas excessive Se induces metabolic imbalance and oxidative stress. Overall, moderate Se fertilizer (1.00 mg/L) can significantly enhance the growth and quality of blueberries, while excessive selenium may have adverse effects.

## 1. Introduction

Selenium (Se) plays a vital role as a trace mineral in some plants [1]. Selenium regulates plant physiological and biochemical functions, acts as an antioxidant, and enhances plant tolerance to multiple abiotic stresses such as heavy metals, salinity-alkalinity, and drought [2,3]. Its effects are concentration-dependent: appropriate doses are beneficial, while excessive doses are harmful [4]. Soil Se concentration exhibits significant variability in China, with over half of the regions classified as Se-deficient or low-Se [5,6]. Hence, investigating the proper use of Se establishes a foundation for producing high-quality Se-enriched products.

The sweet and sour flavor of blueberries is primarily determined by the accumulation and metabolism of carbohydrates and organic acids [7]. Selenium additionally serves a crucial function in facilitating growth, providing antioxidant effects, enhancing stress resistance, and improving disease resistance in plants [8]. Selenium supports plant development and improves the overall quality of fruits when applied to fruit trees [9]. Fruits contain abundant nutrients, including proteins and amino acids, and selenium contributes significantly to improving the quality of blueberries [10]. Appropriate levels of Se application in fruit trees have been shown to significantly increase the soluble solids present in the fruit [11]. The use of Se fertilizer can significantly improve several quality attributes of winter jujube, such as soluble sugars, vitamin C, soluble solids, and total flavonoid content [12]. At present, studies examining the impact of selenium application on improving fruit quality remain relatively limited.

Selenium is naturally present in two main forms: organic and inorganic, while Se in soil is categorized into elemental selenium (Se^0^), selenides (Se^2−^), selenites (Se^4+^), and selenates (Se^6+^) [13]. In addition, Se treatment has a significant positive effect on the contents of total sugars, reducing sugars, soluble solids, as well as the elements P, Cu, Mn, and Mg in grapes [14]. In addition, applying selenium at suitable levels can promote plant growth, effectively alleviate heavy metal stress, and inhibit the accumulation of heavy metals within the plant [15]. Se can also alleviate drought stress in plants [16]. Moderate amounts of Se have beneficial effects on plants, while high concentrations of Se can be toxic [17]. Therefore, investigating the effects of appropriate Se concentrations on plant physiology and metabolic pathways is significant for guiding the application of Se fertilizers in blueberries.

Current research on the effects of selenium on blueberry (*Vaccinium corymbosum* L.) remains limited, and the underlying mechanisms of selenium action have not been fully clarified in terms of agronomic traits, antioxidant systems, and leaf metabolomics. Specifically, the following knowledge gaps still exist: (1) There is a lack of systematic concentration-gradient research on the “moderate-promotion and high-inhibition” effect of Se in blueberry; (2) How does Se regulate sugar metabolism, organic acid metabolism, and volatile compound metabolism in blueberry? (3) The relationship between Se-induced changes in leaf metabolome and antioxidant physiological indicators has not been integrated in blueberry. This study uses blueberry (Brigitta) as the experimental material and investigates the impacts of varying Se levels on blueberry growth and absorption of mineral elements, agronomic traits, antioxidative indicators, and leaf metabolites, providing a scientific basis to produce Se-enriched blueberries. This study proposes the following research hypotheses: (1) Moderate application rates of selenium (Se) fertilizer contribute to both improved growth and quality in blueberry plants, while excessive levels of Se fertilizer may suppress the growth of blueberry plants; (2) Moderate concentrations of Se fertilizer may increase plant growth and development by mediating plant’s own metabolism and activating the antioxidant system, while excessive concentrations can have negative effects; (3) Different concentrations of selenium fertilizer regulate the flavor compounds of blueberries by affecting their sugar metabolism, acid metabolism, and volatile substances.

## 2. Results

### 2.1. Se Concentration Influence on Agronomic Traits of Blueberries

From the result in Table 1, the Se2 treatment demonstrated the most significant improvement in the agronomic traits of blueberry. In comparison to the control group (CK), the blueberry plants showed increases in several traits. These included greater plant height, thicker stems, more leaves, and higher weights for both fresh and dry leaves and stems, with an increase of 60.2%, 61.9%, 36.1%, 34.6%, 46.3%, 88.0% and 80.5% under the Se2 treatment, respectively (*p* < 0.05) (Table 1). Nonetheless, when compared to Se2, the blueberry plants under Se4 treatment experienced decreases in several traits. Specifically, plant height dropped by 32.6%, stem thickness by 36.3%, leaf number by 21.4%, leaf fresh weight by 41.9%, leaf dry weight by 43.5%, stem fresh weight by 30.0%, and stem dry weight by 23.6%. These reductions show a notable difference in performance under the different treatments. (Table 1).

### 2.2. The Effect of Different Se Concentrations on Nutrient Absorption in Leaf of Blueberries

**Figure 1:**
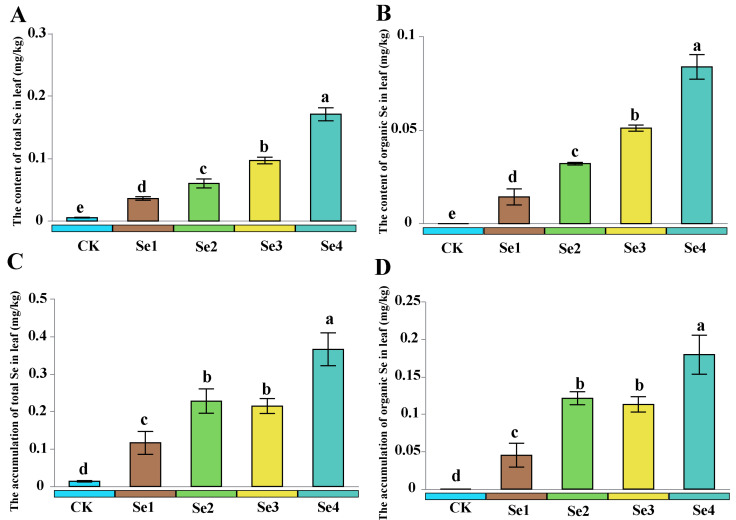
The effect of different selenium fertilizer concentrations on selenium components in leaves. Different lowercase letters indicate significant differences according to Duncan’s test (*p* < 0.05). (**A**): the content of total Se in leaf; (**B**): the content of organic Se in leaf; (**C**): the accumulation of total Se in leaf; (**D**): the accumulation of organic Se in leaf. CK, 0 mg/L sodium selenite; Se1, 0.50 mg/L; Se2, 1.00 mg/L; Se3, 2.00 mg/L; Se4, 3.00 mg/L.

Compared to the CK, the total contents of nitrogen (N), total contents of phosphorus (P), and total contents of potassium (K) in blueberry increased by 48.1%, 15.9%, and 14.3% under the Se2 treatment (Table 2). However, the contents of Total N and Total K decreased by 18.1% and 13.0% under the Se4 treatment, in comparison to CK (Table 2). Similarly, the nutrient accumulation of Total N, Total P, and Total K in blueberries increased by 116%, 69.5%, and 66.7% under the Se2 treatment compared to CK, respectively (Table 2). In contrast, the nutrient accumulation of Total N and Total K in blueberries decreased by 32.5% and 28.1% under the Se4 treatment compared to CK, respectively (Table 2). With the rise in selenium fertilizer concentration, there was a clear increase in the selenium levels found in the leaves (Figure 1). In comparison to CK, the content and accumulation of total Se rose dramatically under the Se4 treatment, showing increases of 2300% and 2514%, respectively (Figure 1). In addition, the content and accumulation of organic Se increased by 538% and 298% under the Se4 treatment, compared to Se1.

### 2.3. Se Concentration Effects on Chlorophyll and Antioxidative Indicators in Leaf of Blueberries

**Figure 2:**
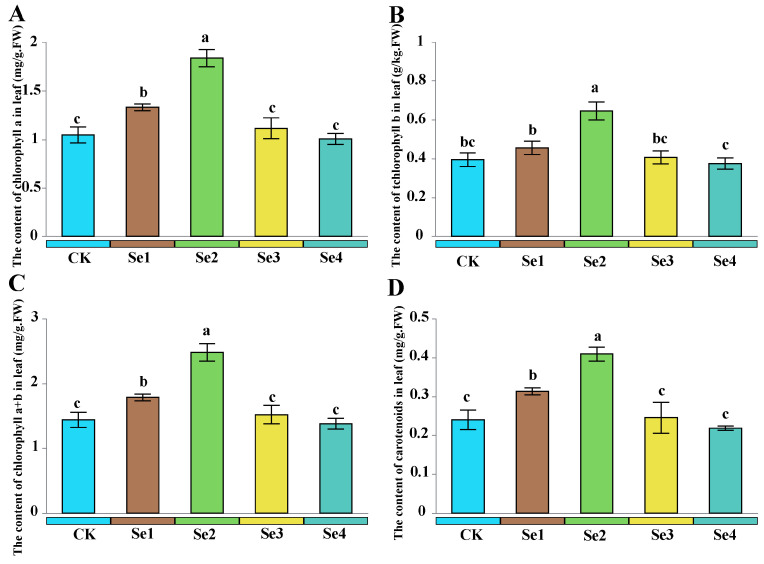
The effect of different selenium fertilizer concentrations on chlorophyll components in leaves. Different lowercase letters indicate significant differences according to Duncan’s test (*p* < 0.05). (**A**): the content of chlorophyll a in leaf; (**B**): the content of chlorophyll b in leaf; (**C**): the content of a + b in leaf; (**D**): the content of carotenoids in leaf. CK, 0 mg/L sodium selenite; Se1, 0.50 mg/L; Se2, 1.00 mg/L; Se3, 2.00 mg/L; Se4, 3.00 mg/L.

According to Figure 2, the application of Se fertilizer affected the chlorophyll content of leaves in blueberries. An appropriate concentration of selenium fertilizer enhanced the chlorophyll content in blueberries, while a high concentration of Se fertilizer reduced the chlorophyll content in blueberries (Figure 2). Compared to CK, the levels of chlorophyll a, b, a + b, and carotenoids all showed an increase by 76.9%, 66.7%, 72.2%, and 70.8% under the Se2 treatment (Figure 2). However, the content of chlorophyll a, b, a + b, and carotenoids was reduced by 45.7%, 43.1%, 44.4%, and 46.4% under the Se4 treatment compared to the Se2 (Figure 2).

The activities of the enzymes SOD, CAT, and POD showed notable increases by 43.5%, 43.0%, and 30.4% under the Se2 treatment in comparison to the control group (Table 3). Meanwhile, the osmoregulatory substances of soluble sugar, soluble protein, and proline (Pro) increased by 15.3%, 8.24%, and 30.1% under the Se2 treatment in relation to the CK group. In addition, there were also increases in the levels of MDA, H_2_O_2_, and O^2−^ by 27.9%, 67.7%, and 4.54% under the Se2 treatment. Compared to the Se2, the SOD, CAT, and POD activities experienced a decrease by 45.6%, 20.9%, and 64.4% under the Se4 treatment (Table 3). Besides, the contents of MDA, H_2_O_2_, O^2−^, SS, SP, and Pro were decreased by 17.7%, 13.4%, 5.46%, 8.92%, 23.91%, 14.12% under the Se4 treatment, compared to the Se2 (Table 3).

### 2.4. Se Concentration Effects on Metabolism in Leaf of Blueberries

**Figure 3:**
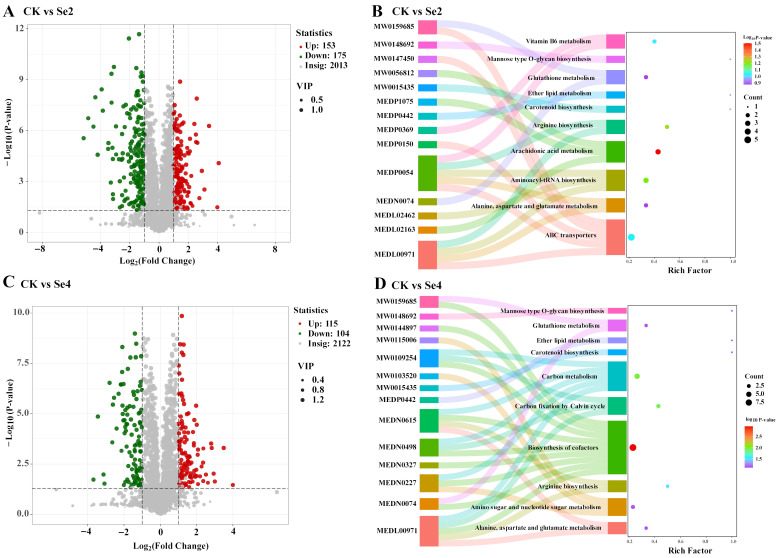
The volcanic map (**A**,**C**) and enrichment analysis (**B**,**D**) under different selenium fertilizer concentrations. (**A**,**B**): CK (0 mg/L) vs. Se2 (1 mg/L); (**C**,**D**): CK (0 mg/L) vs. Se4 (3 mg/L).

Through metabolomic analysis, the primary metabolic products in the leaves are amino acids, organic acids, and benzenoids under different Se concentration treatments (Figure S1A,C). Additionally, the OPLS-DA analysis results revealed that both moderate and high concentrations of selenium fertilizer had a significant impact on the composition of leaf metabolites when compared to the control group (CK), with a significance level of *p* < 0.05. (Figure S1B,D). The findings from the volcano plot revealed that there were notable changes in the metabolites of the leaves following selenium fertilizer treatment (Figure 3A,C). Compared to the CK, there were 328 differential metabolites (up: 153; down: 175) in the leaf with Se2 treatment (1 mg/L), while only 219 differential metabolites (up: 115; down: 104) were found in the leaf with Se4 treatment (3 mg/L) (Figure 3A,C). Enrichment analysis revealed that the differential metabolites were predominantly linked to metabolic pathways like aminoacyl-tRNA biosynthesis, arachidonic acid metabolism, and ABC transporters during the Se2 treatment (Figure 3B). In addition, enrichment analysis showed that differential metabolites mainly focused on nucleotide sugar metabolism, amino sugar metabolism, metabolic pathways, carbon metabolism, and biosynthesis of cofactors under Se4 treatment (Figure 3D).

**Figure 4:**
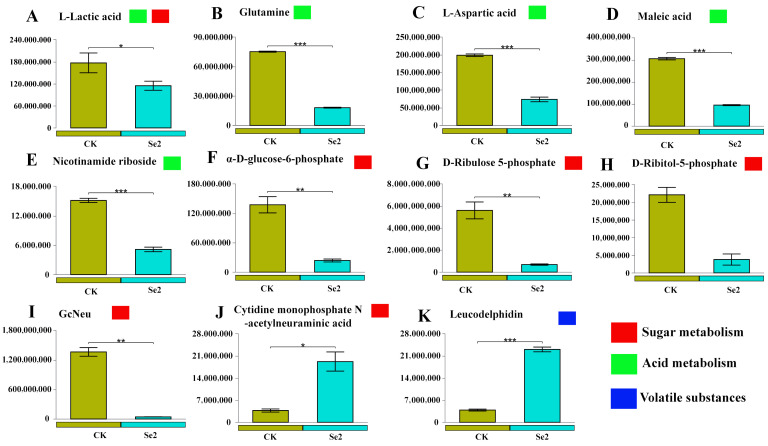
The differential metabolites (sugar metabolism, acid metabolism, and volatile substances) between different treatments (CK, 0 mg/L vs. Se2, 1 mg/L). * *p* < 0.05, ** *p* < 0.01, *** *p* < 0.001. (**A**): L-Lactic acid; (**B**): Glutamine; (**C**): L-Aspartic acid; (**D**): Maleic acid; (**E**): Nicotinamide riboside; (**F**): α-D-glucose-6-phosphate; (**G**): D-Ribulose 5-phosphate; (**H**): D-Ribitol-5-phosphate; (**I**): GcNeu; (**J**): Cytidine monophosphate *N*-acetylneuraminic acid; (**K**): Leucodelphidin.

**Figure 5:**
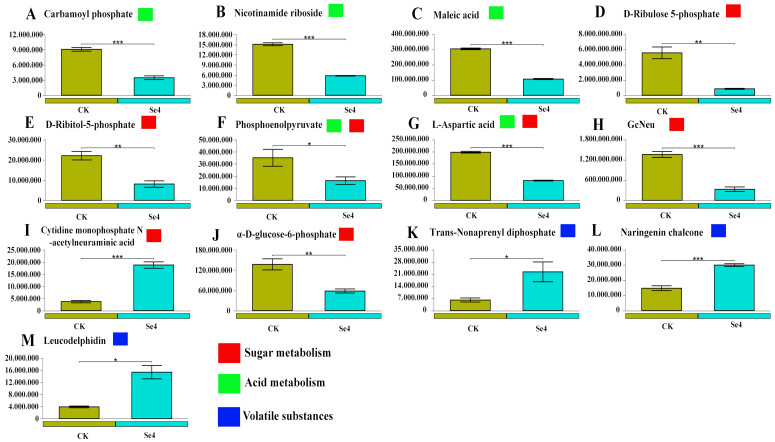
The differential metabolites (sugar metabolism, acid metabolism, and volatile substances) between different treatments (CK, 0 mg/L vs. Se4, 3 mg/L). * *p* < 0.05, ** *p* < 0.01, *** *p* < 0.001. (**A**): Carbamoyl phosphate; (**B**): Nicotinamide riboside; (**C**): Maleic acid; (**D**): D-Ribulose 5-phosphate; (**E**): D-Ribitol-5-phosphate; (**F**): Phosphoenolpyruvate; (**G**): L-Aspartic acid; (**H**): GcNeu; (**I**): Cytidine monophosphate *N*-acetylneuraminic acid; (**J**): α-D-glucose-6-phosphate; (**K**): Trans-Nonaprenyl diphosphate; (**L**): Naringenin chalcone; (**M**): Leucodelphidin.

Subsequently, subset analysis was performed for specific functional categories (sugar metabolism, organic acid metabolism, and volatile substances) based on KEGG pathway annotation. Compared to the control group, there were 11 (2 upregulated and 9 downregulated differential metabolites) and 13 (4 upregulated and 9 downregulated differential metabolites) differential metabolites in the Se2 and Se4 treatments, respectively (Figure 4 and Figure 5). To be more precise, the content of L-Lactic acid (sugar metabolism, acid metabolism), Glutamine (acid metabolism), L-Aspartic acid (acid metabolism), Maleic acid (acid metabolism), Nicotinamide riboside (acid metabolism), α-D-glucose-6-phosphate (sugar metabolism), D-Ribulose 5-phosphate (sugar metabolism), D-Ribitol-5-phosphate (sugar metabolism), and N-glycolylneuraminic acid (sugar metabolism) were decreased by 35.1%, 76.0%, 62.9%, 68.8%, 66.0%, 82.7%, 87.7%, 82.8%, and 96.8% under the Se2 treatment (1.0 mg/L), in comparison to CK (Figure 4A–I). These differential metabolites are primarily involved in glycolysis/gluconeogenesis (α-D-glucose-6-phosphate, phosphoenolpyruvate), pentose phosphate pathway (D-ribulose 5-phosphate, D-ribitol-5-phosphate), and tricarboxylic acid (TCA) cycle-related organic acids (L-lactic acid, maleic acid, L-aspartic acid). However, the content of Cytidine monophosphate *N*-acetylneuraminic acid and Leucodelphidin increased by 419% and 495%, indicating activation of phenylpropanoid/flavonoid pathway (Figure 4J,K). Compared to moderate-concentration Se fertilizer (1.00 mg/L), the differential metabolites in the leaves treated with high-concentration Se fertilizer (3.00 mg/L) exhibited similar variation (Figure 4 and Figure 5). With high concentrations of Se fertilizer addition, the differential metabolic content of sugars and acids significantly decreased, while the content of volatile substances increased markedly (Figure 5). Moreover, most differential metabolites (sugar metabolism, acid metabolism) showed a significant negative correlation with physiological indicators, while only volatile compounds exhibited a positive correlation (Figure S2). In addition, under moderate concentrations of Se fertilizer treatment (1.00 mg/L), differential metabolites showed a significant correlation with leaf nutrients (Figure S2A). In contrast, under high concentrations of Se fertilizer treatment (3.00 mg/L), the differential metabolites were significantly correlated with total selenium (Total Se), organic selenium (Organic Se), and Total K content (Figure S3).

## 3. Discussion

**Figure 6:**
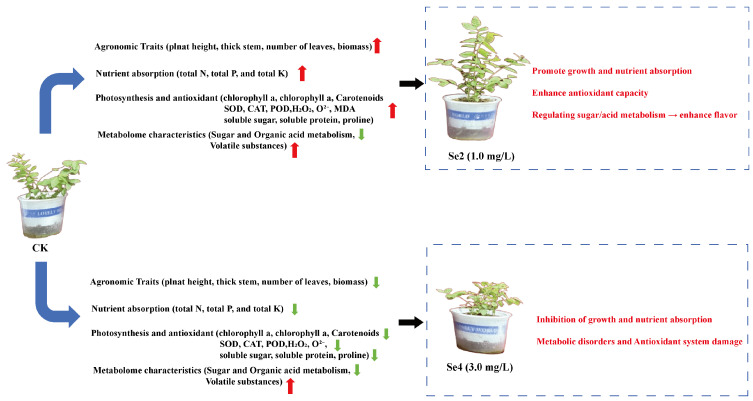
Schematic diagram of the dual effects of CK (0 mg/L) to moderate (Se2, 1.00 mg/L) vs. high Se (Se4, 3.00 mg/L) concentrations on growth, antioxidant defense, and leaf metabolism blueberry. Red arrows represent ascending. Green arrows represent descending.

Comprehensive results of this study indicated that selenium fertilizer had a “ moderate promotion and high-inhibition” effect on blueberries (*Vaccinium corymbosum* L. ‘Brigitta’), with 1.0 mg/L determined as the optimal concentration (Figure 6). Under moderate Se supply (1.0 mg/L, Se2), Se acts as a beneficial element. It promotes plant growth (plant height, stem diameter, leaf number, and biomass), enhances the absorption of macronutrients (N, P, K), and increases photosynthetic pigment content. Moreover, moderate Se upregulates the activities of antioxidant enzymes (SOD, CAT, POD) and osmoprotectants (soluble sugar, soluble protein, proline), thereby avoiding excessive ROS accumulation. Metabolomically, moderate Se downregulates certain intermediates of sugar and organic acid metabolism (e.g., α-D-glucose-6-phosphate, L-lactic acid, maleic acid). In contrast, high Se concentration (3.0 mg/L, Se4) exhibits an inhibitory effect. It suppresses growth and nutrient acquisition, decreases chlorophyll content and antioxidant enzyme activities, and induces severe oxidative stress (elevated MDA, H_2_O_2_, O_2_^−^). Metabolomic analysis further reveals that excessive Se disrupts primary metabolism, including nucleotide sugar metabolism, carbon metabolism, and cofactor biosynthesis, leading to a general decline in sugar-and acid-related metabolites while abnormally elevating some volatile substances.

### 3.1. Se Concentration Effects on Nutrient Absorption and Agronomic Traits of Blueberries

Recent research has indicated that using Se fertilizer can enhance seed germination and improve crop yield and quality [5,18]. In this study, the application of Se fertilizer at moderate concentrations (≤1 mg/L) can significantly promote the growth of blueberries, while excessively high concentrations of Se fertilizer have been found to exert inhibitory effects (Table 1). This may be because Se participates in the synthesis of amino acids within plants, which is beneficial for their physiological metabolism [19]. Moreover, using the right amount of selenium fertilizer can boost nutrient uptake in plants, leading to improved growth [20,21]. In our research, the use of selenium fertilizer at an appropriate concentration greatly enhances the absorption and accumulation of mineral nutrients in blueberry leaves (Table 2). Other studies have shown that using selenium fertilizer at suitable concentrations can noticeably improve both the yield and quality of various crops [22,23]. However, we also observed that applying high concentrations of Se fertilizer (greater than 1 mg/L) tends to inhibit crop growth and the absorption of mineral nutrients (Table 1 and Table 2). When the content of Se-containing amino acids is excessively high, it influences the production of functional proteins while also hindering plant development [24]. Elevated selenium levels can impede the absorption of essential nutrients and accumulation of phosphorus in crops [25,26]. It can be concluded from this study that compared with CK, the contents of total selenium and organic selenium were significantly increased under Se4 treatment, rising by 2300% and 2514%, respectively (Figure 1). Studies have demonstrated that when excessive selenium accumulates in plants, the plants can alleviate stress via mechanisms including methylation volatilization, ABC transporters and vacuolar compartmentalization [27,28].

### 3.2. Se Concentration Effects on Chlorophyll Components and Antioxidative Indicators in Leaf of Blueberries

Selenium helps maintain the stability of cell membranes and boosts photosynthesis in plants. Its presence is vital for promoting overall plant health and function, making it an essential nutrient in agriculture [29,30]. As the concentration of exogenous selenium increased, there was a notable upward trend in the chlorophyll components of the blueberry plants (Figure 2). Some studies have demonstrated that Se influences the synthesis of chlorophyll in plants indirectly by exerting its antioxidant effects, promoting the absorption of mineral elements, and regulating enzyme activity [31]. Additionally, Se regulates the biosynthesis of porphyrins by interacting with sulfhydryl-containing enzymes, and porphyrins are key substances for chlorophyll formation [32]. Se fertilizers can promote the reconstruction of the submicroscopic structure of chloroplasts, reorganize the structures of thylakoids and stroma, increase the content of chlorophyll and carotenoids, and enhance the photosynthesis of plants [33,34]. Selenium can enhance the activities of Rubisco and other enzymes related to the Calvin cycle, thereby indirectly facilitating chlorophyll retention [35].

Numerous studies have shown that selenium boosts resilience against stress, enhances crop quality, and aids in protein synthesis [1,36,37]. Using selenium fertilizer at suitable concentrations boosts the activity of antioxidant enzymes and increases the levels of osmotic regulatory substances in the leaves (Table 3). Moderate selenium can also help mitigate salt stress by regulating SOD, POD, and CAT and osmotic metabolic processes [38,39]. Low levels of ROS act as signaling molecules and may enhance the overall defense capacity of plants [40]. However, applying high concentrations of exogenous selenium fertilizers tends to lead to a decrease in the levels of antioxidant enzymes in the leaves compared to CK (Table 3). Osmotic regulation lowers cellular osmotic potential and maintains water potential balance as well as the stability of the cell protoplasmic membrane by promoting the accumulation of osmotic regulating substances (proline, soluble sugars, etc.) [41]. Excessive selenium can be toxic to plants, as it promotes the buildup of superoxide radicals. This results in oxidative stress that harms cell membranes, organelles, and even DNA [42,43]. In our study, we found that applying selenium fertilizer significantly raised the levels of soluble sugars, soluble proteins, and proline in the leaves (Table 3). Se reduces the accumulation of ROS by promoting the accumulation of proline and soluble sugars, enhancing the expression of related genes, protecting cell membranes from damage, and maintaining normal physiological functions [44]. Compared with Se2 treatment, the reductions in the contents of antioxidant enzymes and osmotic adjustment substances in leaves under Se4 treatment (Table 3) might be due to excessive selenium accumulation exceeding the tolerance threshold of blueberry plants, leading to a systematic decline in all physiological activity indicators [45].

### 3.3. The Influence of Varying Selenium Concentrations on Metabolism in Blueberry Leaves

The metabolism of nutritional components in plants is primarily manifested in several aspects including energy conversion, protein synthesis, regulation of synthesized compounds, and the metabolism of plant chlorophyll with Se addition [46]. In our study, Se significantly affects the differential metabolites in blueberry leaves, with a greater number of differential metabolites observed at moderate concentrations of Se fertilizer (Figure 3). As a beneficial element, moderate Se activates multiple signaling pathways and metabolic networks, including primary carbon metabolism, amino acid metabolism, antioxidant defense, and secondary metabolism [4]. Se supplementation not only increases the Se content in plants but also influences the accumulation of secondary metabolites in plants, such as terpenes and phenolic compounds [47]. Enrichment analysis revealed that the differential metabolites were predominantly linked to metabolic pathways like aminoacyl-tRNA biosynthesis, arachidonic acid metabolism, and ABC transporters during the Se2 treatment (Figure 3). These pathway enrichments indicated that moderate selenium treatment could enhance the protein synthesis capacity of leaves, activate the biosynthesis of defensive substances, and participate in the vacuolar transport of secondary metabolites such as flavonoids, thereby promoting the growth of blueberry [48,49,50]. Additionally, selenium fertilizer contributes positively to enhancing crop quality, notably by increasing the amino acid content in tea leaves [51]. However, high selenium causes loss of protein function and inhibition of protein synthesis, further inducing metabolic disorders and pathway suppression [52].

Sugar metabolism, acid metabolism, and volatile compounds are crucial substances that determine the flavor quality of blueberries [53]. In our study, selenium fertilizer application significantly decreased the metabolites associated with sugar metabolism and acid metabolism in blueberry leaves, and simultaneously increased the content of volatile compounds. (Figure 4 and Figure 5). The major reason is that plants can avoid the excessive formation of ROS by reducing the accumulation of intermediates in the energy metabolism under external stress [54]. In addition, Se fertilizer may promote the biosynthesis of volatile substances by activating secondary metabolic pathways [55]. Many studies indicate that applying selenium fertilizers can help slow the breakdown of organic acids, boost the levels of soluble solids, and preserve the flavor characteristics of fruits [56,57]. The flavor metabolites in our study exhibited a significant correlation with antioxidant indicators, and this correlation was even closer at appropriate Se concentrations (Figures S2 and S3). Selenium promotes the expression of antioxidant-related genes in mustard (SOD, CAT, POD, and PAL) [58]. Furthermore, studies have found that nano-selenium activates glutathione metabolism in fruits. This activation could provide a molecular basis for its improved antioxidant capacity, showcasing the benefits of selenium at the nano level in enhancing fruit health [59]. However, high concentrations of Se fertilizer may cause damage to cell membrane activity, affecting the metabolic balance of fruits [60].

## 4. Materials and Methods

### 4.1. Study Site Characteristics and Experimental Design

In this study, seedlings of the blueberry cultivar (Brigitta) were selected as experimental materials (Anhui Science and Technology University, Chuzhou, Anhui Province, China), and a pot experiment was carried out to simulate substrate culture conditions. In this study, selenium applied to the pots was supplied in the form of sodium selenite (Na_2_SeO_3_) (purity ≥ 99%, Guangdong Chemical Products Sales Company, Guangzhou, China). The concentrations of sodium selenite added were 0 mg/L (CK), 0.50 mg/L (Se1), 1.00 mg/L (Se2), 2.00 mg/L (Se3), and 3.00 mg/L (Se4), resulting in a total of five treatments with five replicates (n = 5). The substrate used was a special substrate for blueberry cultivation, and its basic physicochemical properties are as follows: pH 5.25, organic matter 53.6 g/kg, alkaline hydrolyzable nitrogen 372 mg/kg, available phosphorus 79.9 mg/kg, available potassium 897 mg/kg, and total selenium 0.65 mg/kg. The application rates of N, P_2_O_5_, and K_2_O were 0.20 g/kg of air-dried blueberry cultivation substrate, and each plastic pot (diameter 15.8 cm, height 10.3 cm) contained 200 g of blueberry substrate. Nitrogen was supplied in the form of urea, phosphorus was provided as KH_2_PO_4_, and the remaining potassium was supplemented with K_2_SO_4_ to reach the intended K_2_O level. After 60 days of rooting culture, robust seedlings with a plant height of 8–10 cm were uniformly selected and transplanted into pots. Blueberry seedlings were transplanted into the substrate in mid-June, and selenium fertilizer treatment was applied in mid-July. Sodium selenite (Na_2_SeO_3_) was applied via root irrigation every 10 days, with a volume of 10 mL per pot each time, for a total of 4 times.

### 4.2. Sampling and Analysis

Before sample harvesting, agronomic traits (plant height, stem diameter, number of leaves) were measured for plants under different treatments. Samples were separated into two portions: one portion of fresh samples was used to determine chlorophyll content, physiological indicators, and metabolic products, while the other part was used for the determination of Se content. All measurements were performed on five independent biological replicates per treatment (5 × 5).

Determination of chlorophyll and carotenoids: Fresh leaves were extracted using a solvent (80.0% acetone) and kept in the dark at room temperature for 24 h. Absorbance was subsequently recorded at wavelengths of 665 nm, 649 nm and 470 nm using a multimode reader platform (Tecan, Spark 10, Mannedorf, Switzerland) [61]. The contents of chlorophyll a, chlorophyll b, and carotenoids are all expressed as milligrams per gram of fresh weight (mg/g FW).

Determination of plant nutrients: The leaf samples of blueberry were subjected to digestion using a mixed acid of nitric acid and perchloric acid (10:1, *v*/*v*), followed by the determination of total Se and organic Se content using atomic fluorescence spectroscopy [62]. Following the grinding of the various plant tissues, the contents of total nitrogen (N), total phosphorus (P), and total potassium (K) were measured. Total nitrogen (N) was quantified using the H_2_SO_4_–H_2_O_2_ digestion–Kjeldahl method, total phosphorus (P) was determined by the vanadium–molybdenum yellow colorimetric method, and total potassium (K) was analyzed using flame photometry. These techniques were based on earlier research [63].

Determination of plant physiological indicators: the activities of superoxide dismutase (SOD), catalase (CAT), and peroxidase (POD) were evaluated using the nitro blue tetrazolium (NBT) method, ammonium molybdate method, and guaiacol method, respectively. H_2_O_2_, superoxide anion (O^2−^), malondialdehyde (MDA), and proline (Pro) contents were measured with the titanium sulfate colorimetric method, the α-naphthylamine colorimetric method, the thiobarbituric acid colorimetry method, and the acid ninhydrin colorimetric method. Soluble sugar (SS) was determined by anthrone colorimetry, and soluble protein (SP) was determined by Coomassie blue colorimetric assay. All methods are referenced to [64].

### 4.3. Metabolomics Determination

For metabolomic analysis, five biological replicates (n = 5) were selected from three treatments (CK, Se2, and Se4). For each plant sample, 100 mg of tissue was ground in liquid nitrogen and transferred into an EP tube. Then, 500 μL of a solution containing 0.10% formic acid and 80.0% methanol in water was added. The mixture was vortexed, incubated in an ice bath, and allowed to stand for 5 min. Subsequently, the mixture was transferred to a centrifuge tube and centrifuged at 15,000 rpm and 4 °C for 10 min. The supernatant was carefully collected and diluted with an appropriate volume of mass spectrometry–grade water. The solution was then transferred to a centrifuge tube again and centrifuged at 15,000 rpm and 4 °C for another 10 min. Finally, the supernatant was carefully collected for injection and subsequent analysis. All untargeted metabolomics sequencing procedures were performed by Wuhan MetWare Biotechnology Co., Ltd. (Wuhan, China).

Raw data were analyzed with Progenesis QI v2.3 software from Nonlinear Dynamics, based in Newcastle, UK, and this software was utilized for the preprocessing of the raw data, including noise filtering, deconvolution, peak intensity calibration, as well as qualitative and quantitative analyses of the obtained spectra. Multivariate statistical analysis was carried out with SIMCA-P 14.1 software. We conducted Student’s *t*-test and analyzed fold changes. To visualize the results, a univariate statistical approach was used to generate volcano plots, and this was done using R software (version 4.2.3). The criteria for screening differential compounds were set at *p* < 0.05 and VIP > 1. Subsequently, subset analysis was performed for specific functional categories (sugar metabolism, organic acid metabolism, and volatile substances) based on KEGG pathway annotation. Hierarchical clustering of differential metabolites with higher VIP values was performed using R software, and volcano plots were generated, facilitating the selection of differential metabolites. Correlation analysis was carried out using Pearson correlation coefficients to assess the linear relationships between pairs of metabolites. Additionally, pathway enrichment analysis utilized KEGG IDs from the differential metabolites to identify the metabolic pathways that showed significant enrichment among those with notably different expression levels (*p* < 0.05). All of these analyses were completed in the Metware cloud platform (http://cloud.metware.cn/).

### 4.4. Statistical Analysis

In this experiment, we applied variance analysis by R software, specifically version 4.2.3. Duncan’s test, with a significance level of *p* < 0.05, was used for multiple comparisons. Additionally, Version 4.2.3 of the R software was employed to analyze all data and create plots. We explored the relationship between differential metabolites and physiological indicators through correlation analysis with R (corrplot package).

## 5. Conclusions

The findings of this study suggest that the proper use of exogenous selenium fertilizer can greatly boost the growth and development of blueberries. It demonstrates moderate Se (1.00 mg/L) application improves blueberry growth while excessive Se (3.00 mg/L) suppresses growth is confirmed, as demonstrated by the agronomic traits and nutrient absorption. In addition, moderate Se (1.00 mg/L) enhances growth by modulating metabolism and activating the antioxidant system, whereas excessive Se (3.00 mg/L) causes negative effects. Moderate Se treatment (1.00 mg/L) increased the activity of antioxidant enzymes and osmoregulatory substances (SS, SP, and Pro) to avoid excessive ROS accumulation in blueberry leaves. However, excessively high selenium concentrations (3.00 mg/L) resulted in a decline in these indicators. Se fertilizer significantly altered the composition of metabolites in blueberry leaves, primarily focusing on amino acids, organic acids, and phenolic compounds. Additionally, applying selenium fertilizer can affect the flavor compounds in blueberries, primarily manifested in the reduction in products from sugar metabolism and acid metabolism, while increasing the content of volatile compounds. Overall, our results support the beneficial role of moderate Se (1.00 mg/L) and the detrimental effects of excessive Se (3.00 mg/L) on blueberry physiology and metabolism. This study offers a scientific foundation for refining selenium fertilization strategies to produce high-quality Se-enriched blueberries.

## Figures and Tables

**Table 1:** Changes in agronomic traits of blueberry under different Se concentrations.

Treatment	Plant Height (cm)	Thick Stem (cm)	Leaf Number	Leaf Fresh Weight (g/Plant)	Leaf Dry Weight (g/Plant)	Stem Fresh Weight (g/Plant)	Stem Dry Weight (g/Plant)
CK	20.5 ± 0.64 d	1.89 ± 0.20 b	86 ± 8.00 d	10.42 ± 1.89 bc	2.59 ± 0.20 bc	2.66 ± 0.25 d	0.87 ± 0.11 d
Se1	25.9 ± 3.01 b	2.12 ± 0.28 b	103 ± 3.06 bc	11.42 ± 2.16 ab	3.22 ± 0.69 ab	3.19 ± 0.31 cd	0.98 ± 0.13 cd
Se2	32.8 ± 1.39 a	3.06 ± 0.34 a	117 ± 3.00 a	14.03 ± 1.58 a	3.79 ± 0.22 a	5.00 ± 0.19 a	1.57 ± 0.08 a
Se3	24.7 ± 2.38 bc	2.13 ± 0.02 b	114 ± 6.00 ab	8.33 ± 0.38 c	2.21 ± 0.14 c	4.36 ± 0.37 b	1.40 ± 0.13 ab
Se4	22.1 ± 0.56 cd	1.95 ± 0.09 b	92 ± 10.00 cd	8.15 ± 0.89 c	2.14 ± 0.16 c	3.50 ± 0.38 c	1.20 ± 0.16 bc

Note: Different lowercase letter indicated significant differences according to Duncan’s test (*p* < 0.05). All values are shown as the mean ± SD. CK, 0 mg/L sodium selenite; Se1, 0.50 mg/L; Se2, 1.00 mg/L; Se3, 2.00 mg/L; Se4, 3.00 mg/L.

**Table 2:** Changes in nutrient absorption of blueberry under different Se concentration.

Treatment	Content			Accumulation		
	Total N (mg/g)	Total P (mg/g)	Total K (mg/g)	Total N (mg)	Total P (mg)	Total K (mg)
CK	19.8 ± 2.59 c	1.83 ± 0.02 c	40.6 ± 1.44 c	51.4 ± 8.11 c	4.75 ± 0.41 c	105.5 ± 11.00 c
Se1	23.3 ± 0.67 b	1.97 ± 0.01 b	42.6 ± 1.90 bc	74.8 ± 14.4 b	6.35 ± 1.35 b	138 ± 33.79 b
Se2	29.3 ± 0.15 a	2.12 ± 0.05 a	46.4 ± 0.15 a	111.0 ± 5.96 a	8.05 ± 0.66 a	176 ± 10.06 a
Se3	20.9 ± 0.81 c	1.98 ± 0.07 b	44.3 ± 0.97 ab	46.2 ± 1.26 cd	4.39 ± 0.30 c	98.2 ± 7.46 c
Se4	16.2 ± 0.14 d	1.83 ± 0.05 c	35.4 ± 2.24 d	34.7 ± 2.86 d	3.92 ± 0.37 c	75.8 ± 9.89 c

Note: Different lowercase letter indicated significant differences according to Duncan’s test (*p* < 0.05). All values are shown as the mean ± SD. CK, 0 mg/L sodium selenite; Se1, 0.50 mg/L; Se2, 1.00 mg/L; Se3, 2.00 mg/L; Se4, 3.00 mg/L.

**Table 3:** Changes in antioxidant enzymes, antioxidants, and osmoregulatory substances of blueberry under different Se concentration.

Treatment	SOD (U/g)	CAT (U/g)	POD (U/g)	MDA (nmol/g)	H_2_O_2_ (nmol/g)	O^2−^ (nmol/g)	SS (mg/g)	SP (mg/g)	Pro (μg/g)
CK	473 ± 6.43 d	1.07 ± 0.02 d	0.56 ± 0.01 c	32.6 ± 0.31 c	20.1 ± 0.96 d	6.83 ± 0.17 bc	11.6 ± 0.24 c	0.85 ± 0.04 bc	10.8 ± 0.24 c
Se1	627 ± 6.21 b	1.11 ± 0.01 d	0.67 ± 0.03 b	37.6 ± 0.91 b	30.9 ± 0.74 b	6.95 ± 0.17 abc	12.6 ± 0.51 ab	0.90 ± 0.02 ab	12.4 ± 0.26 b
Se2	679 ± 4.37 a	1.53 ± 0.02 a	0.73 ± 0.01 a	41.8 ± 1.63 a	33.6 ± 0.75 a	7.14 ± 0.03 a	13.3 ± 0.41 a	0.92 ± 0.03 a	14.0 ± 0.11 a
Se3	521 ± 7.72 c	1.30 ± 0.03 b	0.57 ± 0.03 c	38.7 ± 1.33 b	29.6 ± 0.74 bc	7.01 ± 0.01 ab	12.7 ± 0.51 ab	0.84 ± 0.01 c	12.7 ± 0.52 b
Se4	369 ± 6.03 e	1.21 ± 0.03 c	0.26 ± 0.01 d	34.4 ± 0.69 c	29.1 ± 0.67 c	6.75 ± 0.05 c	12.2 ± 0.43 bc	0.70 ± 0.01 d	12.0 ± 0.40 b

Note: Different lowercase letter indicated significant differences according to Duncan’s test (*p* < 0.05). All values are shown as the mean ± SD. CK, 0 mg/L sodium selenite; Se1, 0.50 mg/L; Se2, 1.00 mg/L; Se3, 2.00 mg/L; Se4, 3.00 mg/L.

## Data Availability

The original contributions presented in this study are included in the article/Supplementary Material. Further inquiries can be directed to the corresponding author.

## Supplementary Materials

The following supporting information can be downloaded at: https://www.mdpi.com/article/10.3390/plants15101532/s1, Figure S1: The material composition analysis (A,C) and OPLS-DA analysis (B,D) under different selenium fertilizer concentrations. (A,B): CK vs. Se2; (C,D): CK vs. Se4; Figure S2: The correlation analysis between antioxidative indicators and differential metabolites (Sugar metabolism, Acid metabolism, and Volatile substances). (A): CK vs. Se2; (B): CK vs. Se4; Figure S3: The correlation analysis between plant nutrients and differential metabolites (Sugar metabolism, Acid metabolism, and Volatile substances). (A): CK vs. Se2; (B): CK vs. Se4.
