# Supplementary material for: Effects of Different Selenium Concentrations on Agronomic Traits, Antioxidant Defense, and Leaf Metabolome in Blueberry (Vaccinium corymbosum L. ‘Brigitta’)
Source: Plants (Basel). 2026 May 17;15(10):1532. doi: 10.3390/plants15101532 (PMC13210566; doi:10.3390/plants15101532)
Supplement: Supplementary file 1 [file plants-15-01532-s001.zip › plants-4310765-supplementary.pdf]

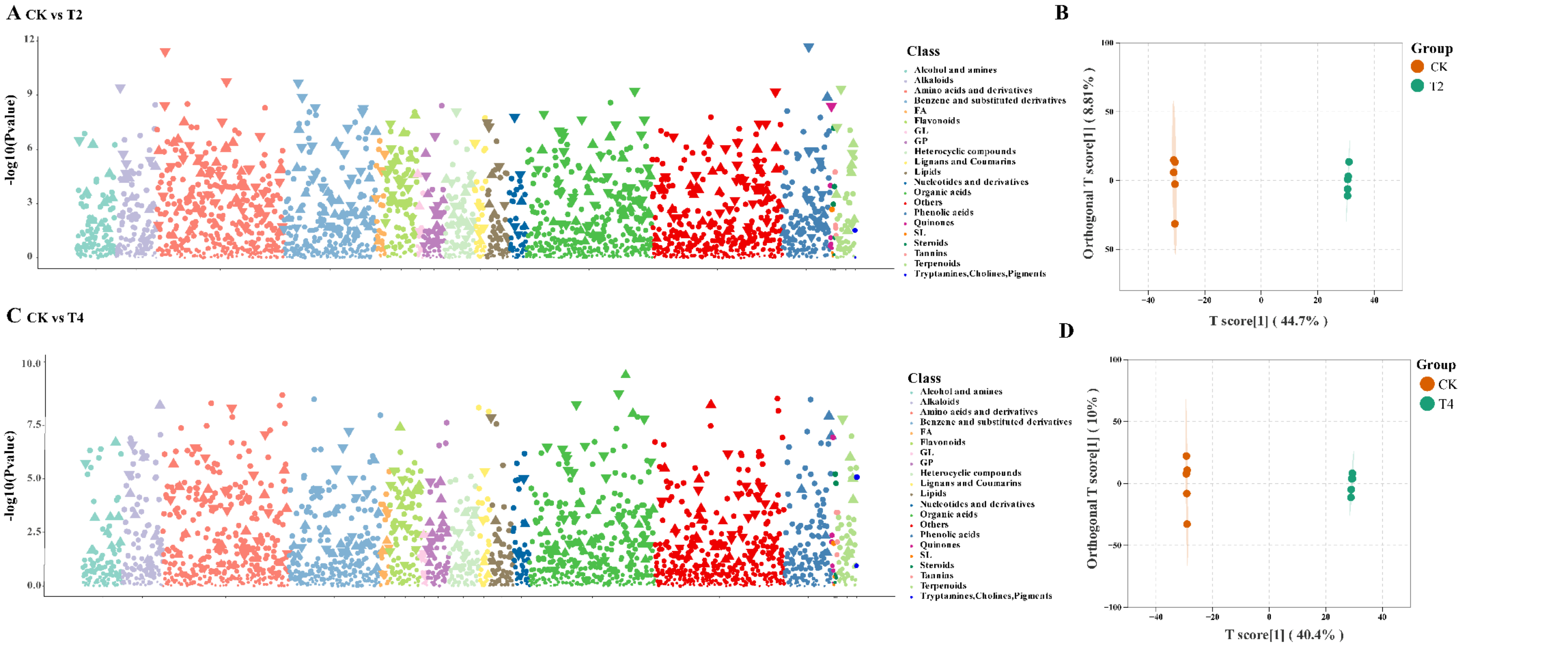

Figure S1 The material composition analysis (A and C) and OPLS-DA analysis (B and D) under different selenium fertilizer concentrations. A and B: CK vs Se2; C and D: CK vs Se4.

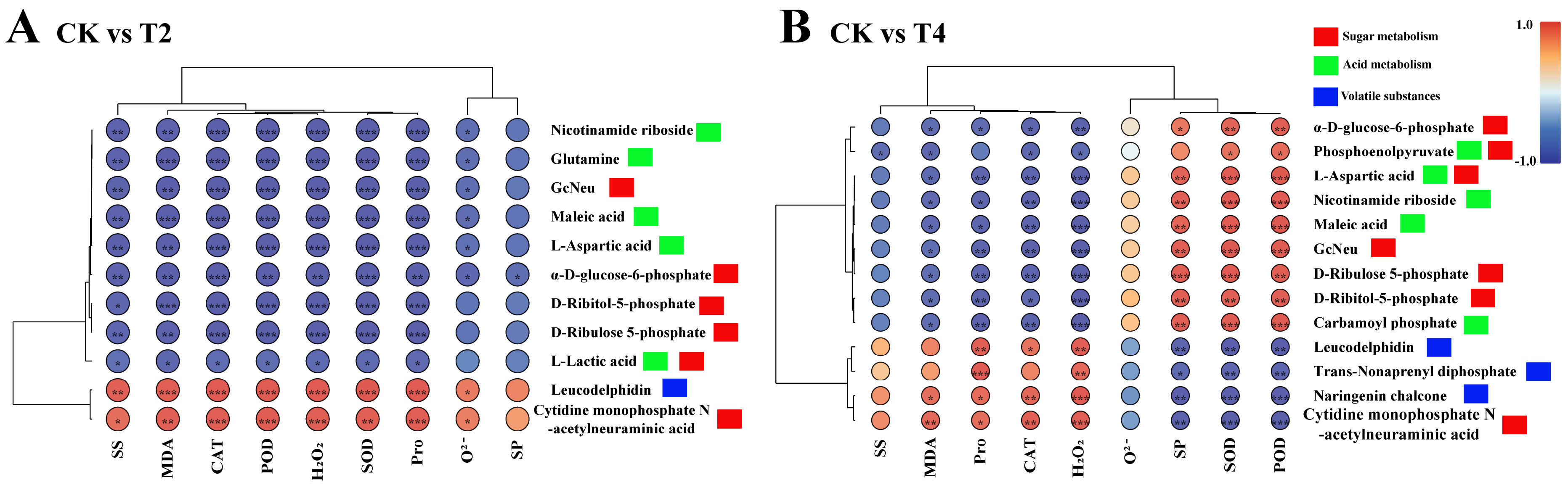

Figure S2 The correlation analysis between antioxidative indicators and differential metabolites (Sugar metabolism, Acid metabolism, and Volatile substances). A: CK vs Se2; B: CK vs Se4.

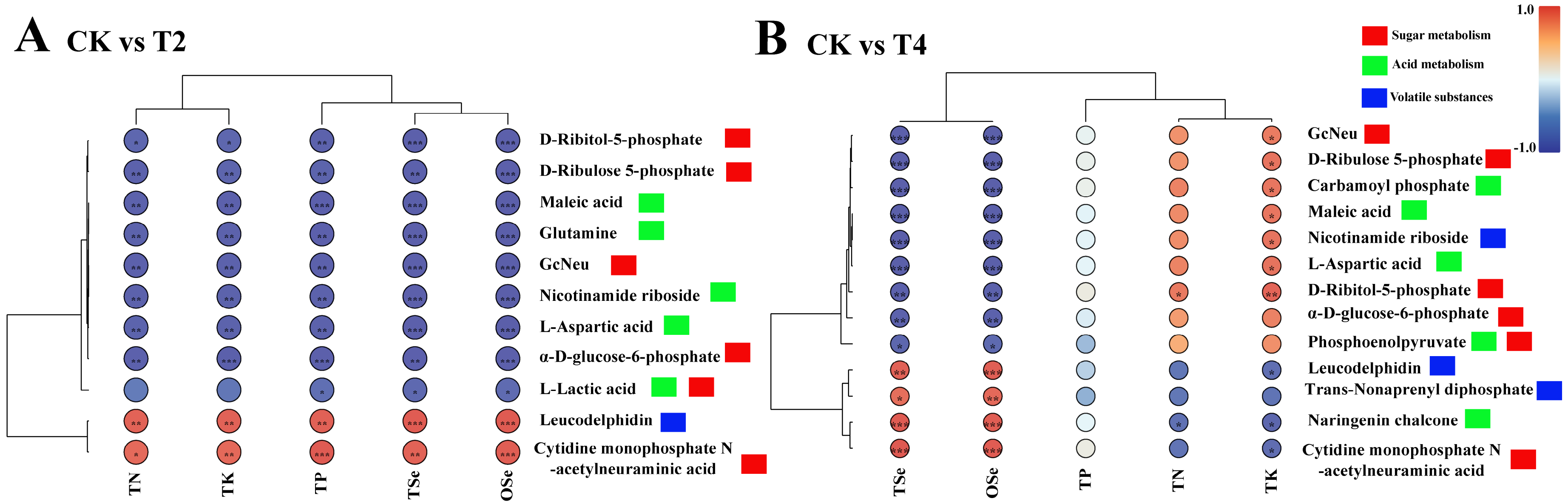

Figure S3 The correlation analysis between plant nutrients and differential metabolites (Sugar metabolism, Acid metabolism, and Volatile substances). A: CK vs Se2; B: CK vs Se4.
